# The Close Relationship Existing between the Veterinary Profession of To-day and Organized Societies for the Prevention of Cruelty to Animals1Presented to the Convention of Humane Societies at Nashville, October, 1897.

**Published:** 1898-01

**Authors:** W. H. Dalrymple

**Affiliations:** Baton Rouge, La.


### THE CLOSE RELATIONSHIP EXISTING BETWEEN THE VETERINARY PROFESSION OF TO-DAY AND ORGANIZED SOCIETIES FOR THE PREVENTION OF CRUELTY TO ANIMALS.[1]

By W. H. Dalrymple, M.R.C.V.S.,
BATON ROUGE, LA.

(Concluded from page 765, December, 1897.)

There is another thought along this line which, if I passed over without allusion, I would feel recreant to duty. I refer to the pain that is often inflicted upon the noblest of our dumb friends as the result of the dictates of *fashion*. There are many people who will thoughtlessly—and I trust I am correct in saying, unknowingly, that is, of the consequent suffering of the animal—require of the veterinarian the performance of some operation, simply because fashion seems to demand it. Relative to this topic, permit me to quote to you the remarks of the eminent British veterinary authority, Dr. George Fleming, in his work on *Surgery*. He says: "Humanity is largely concerned in the humane treatment of animals and in relieving them from pain or distress. All *unnecessary* painful operations are acts of cruelty, and should be discountenanced by the veterinary surgeon. These operations, which, after all, are only fashionable mutilations of perfect animals devised by a morbidly artificial and corrupt taste, should be suppressed, if not by law, at least by the influence of the surgeon." From the quotation just made it may be inferred that this is the opinion and sentiment of many of the most eminent members of the profession to-day; but so long as owners remain the slaves of fads and fashions, and ignorant of the pain these "fashionable mutilations" and other modes of torture, from whatever cause, inflict upon their animals, so long will these unnecessary and painful operations and methods be practised. In other words, the demand for such must be decreased through the better education and enlightenment of the people in those matters, a knowledge of which would greatly preclude the possibility of such requests being made by those of intelligence.

I have, in the foregoing, omitted reference to open and wanton acts of cruelty which could not possibly escape the observation of even the unitiated, and have deemed it more instructive to allude

[1] Presented to the Convention of Humane Societies at Nashville, October, 1897.

to some of the agencies which lead to painful results, but which are liable to escape the notice of those responsible for their production, with, however, no cruel intent.

Before leaving this part of my paper I would like to add a word or two with reference to lameness. It is my opinion that some of the officers or inspectors of our humane societies are not sufficiently familiar with the pathology of this condition, and seem to attach less importance, relatively, to it, and more to other conditions which to the casual observer may appear extremely severe, yet do not, in reality, produce nearly so much pain as that causing an animal to go lame. To illustrate: The driver or owner of an animal may be arrested and fined because of his animal having an abrasion of the skin, or "gall," as it is sometimes termed, below its collar, no larger perhaps than a twenty-five cent piece; yet, on the other hand, fifty or one hundred lame animals may be allowed to pass unheeded, unless in the case of those that are absolutely prostrated and unable to proceed. Now, in the case of the abraded shoulder, there may be extremely slight, if any, pain at all; while in the latter, lameness is never exhibited without pain, unless in the case of deformity resulting probably from some previous injury or pathological condition. It must not be understood that I am in any way trying to exonerate the owner of the animal with the sore shoulder and attach blame to the officer for his vigilance. This is not my object. All indications of cruelty, whether exposed to view or occult, should be dealt with; but I am of the opinion, and that from experience, that lameness in our city and other horses does not receive the attention it merits at the hands of our humane societies for the alleviation of a great deal of suffering of these animals. In continuing the consideration of the grounds upon which I claim that the profession is a most potent factor in the education of the people in the interests of humanity, I have, in the first place, to notice the fitness of the veterinarian, on account of his special course of instruction and training, which familiarizes him with the lower animals in health and in disease. He has made a study of the anatomy, both macroscopical and histological, of the various systems of the equine species, and the differential characteristics exhibited by the other domesticated animals; the physiology or functions of the numerous organs or tissues of the body, including that of movement, nutrition, etc.; dietetics, also, including the chemical analysis of the various alimentary matters, their food-value and their nutritive relations, as well as the quantity and quality required for maintenance, repair, for growth or for work.

He has studied also the various medicinal agents, both mineral and vegetable, and their actions: physiologic, therapeutic, and toxic, and pathology as well, or the study of disease to which the various systems of the animal economy are susceptible, and the most modern prophylactic and therapeutic measures to be adopted for its prevention, control, eradication, or cure; the subject of bacteriology, with its myriad forms of organismal life, their life-histories, the poisonous alkaloids they elaborate, and the antitoxins to antagonize their destructive and deadly effects. Suffice it to say that there is no branch of medical science with which the modern graduate is not familiar in its application to the lower animals, the cause of whose distress and pain cannot be elicited by interrogatories, because the symptoms are entirely objective, and have to be diagnosed by careful investigation and observation.

Possessed of such knowledge, then, can there be any good or just reason to doubt the fitness of the veterinarian as an educator of the people, or as a valuable aid to the work of our societies which are organized for humanity's sake.

As to the various channels through which representatives of the veterinary profession are, or can become instructors or educators in this great and noble cause, I might mention, first, the regular colleges, whose object is to train young men for their life-work along the lines just indicated, who go out into the world equipped with the knowledge of humane methods for the prevention and alleviation of animal suffering. Again, those of us through our connection with State universities and A. and M. colleges have the privilege and opportunity of impressing upon the youthful mind scientific truths bearing upon this subject and the Heaven-born principles of humanity in the care and treatment of live-stock.

The general practitioner, in his daily routine of professional work, has the opportunity to illustrate and explain to his client the superior advantage to be gained by the use of rational and humane methods of treatment, and to denounce the barbarisms that are perpetrated by the ignorant, illiterate, and superstitious. And I am of the opinion that anyone who would stand by and complacently witness the inhuman treatment of a dumb animal by one who is totally ignorant of the true condition, or its proper and legitimate treatment, is an accessory, and is guilty of a wanton act of cruelty.

There can be no doubting the fact, then, I think, that the veterinary profession of to-day is a most important aid in the accomplishment of the great end to which our humane societies are striving; but to strengthen its hands along this line it needs a little more appreciation and encouragement. It must be borne in mind by our people that the day of the old "horse-doctor" is fast nearing its close, and that that worthy, who served his day and generation as best he could according to his light, has been superseded by a profession that is as honorable as it is noble, and which has within its ranks men of as high order of intelligence and education as in any other of the learned professions.

The object of our humane societies is perhaps the noblest under Heaven: preventing, mitigating, or altogether relieving the pain and suffering of those creatures who have not the opportunity nor the power within themselves to do so; and there is no organization which should be so gigantic in its membership or so universal in its power for good. People of all ranks, whether social, political, or religious, should be on its roll, and lend their best efforts to carry on the good work. We must educate ourselves out of the too common idea that the lower animals are mere automatons, and learn to know that they are flesh and blood like ourselves. Let us all adopt the golden rule, and apply it to those dumb servants and companions which the Creator has placed within our care, for our benefit, comfort, and happiness. Then we will have the pleasing satisfaction of knowing that in doing our duty to that noble part of the creation we are performing a duty to the Creator himself.

I will close with two verses from a short poem on "The Hoss," by James Whitcomb Riley:

> "But when I see the beast abused,
> And clubbed around as I've saw some,
> I want to see his owner noosed,
> And jest yanked up like Absolum!
>
> "I love my God, the first of all,
> Then him that perished on the cross;
> And next my wife—and then I fall
> Down on my knees and love the hoss."

---

Nothing is more conducive to bad-looking, ill-fed appearing draught-teams of horses than the habit of American drivers to trot these heavy horses at every opportunity. Many of the sons and dams of these horses were bred for four or five generations to a walking gait, and when driven beyond this pace become hard keepers and unattractive looking animals.

